# Anti-yeast IgY antibodies as a novel biological additive to enhance fermentation and preservation of sugarcane silage

**DOI:** 10.1016/j.vas.2026.100623

**Published:** 2026-03-14

**Authors:** Balieiro G․N․, Rosa C․A․, Engracia Filho J․R․, Budino F․E․L․, Freitas A․W․P․, Moraes J․E․, Pizzolante C․C․

**Affiliations:** a Animal Science Institute, Sao Paulo Agency for Agribusiness Technology–APTA, Department of Agriculture and Food Supply, Ribeirao Preto, SP, Brazil; b Federal University of Minas Gerais, 6627 Antonio Carlos Ave, 31.270-901, Pampulha, Belo Horizonte, Minas Gerais, Brazil; c Pontificia Universidade Catolica do Parana, Curitiba, PR, Brazil

**Keywords:** IgY antibodies, Yeast inhibition, Sugarcane silage, Fermentative losses, Aerobic stability

## Abstract

This study evaluated the effectiveness of a biological silage additive containing IgY antibodies targeting key silage-associated yeasts, aiming to enhance fermentation quality and aerobic stability of sugarcane silage. Sugarcane forage was ensiled with no additive (control), *Lactobacillus buchneri* (5.0 × 10¹⁰ CFU t⁻¹), or IgY at 0, 175, 350, and 700 g t⁻¹ of fresh forage. After 114 days, silages were analyzed for fermentation profile, yeast counts, ethanol concentration, dry matter (DM) recovery, and aerobic stability. IgY at 350 and 700 g t⁻¹ reduced yeast counts below detection limits at silo opening, decreased ethanol production and gas losses, increased DM recovery, and indirectly favored higher lactic and acetic acid concentrations. These treatments remained aerobically stable for over 72 h, with no detectable yeast regrowth or temperature increase. The findings indicate that IgY antibodies effectively limit yeast activity, contributing to improved fermentation patterns and enhanced nutrient preservation. IgY-based additives may represent a biologically derived approach to mitigate fermentative losses in sugarcane silage.

## Highlights

• IgY antibodies specifically targeting yeast suppress yeast growth in sugarcane silage.
• IgY supplementation at 350 g t⁻¹ enhances lactic and acetic acid retention.
• IgY-treated silages show superior dry matter preservation and reduced ethanol production.

## 1 Introduction

Sugarcane (*Saccharum officinarum L*.) is a high-yield tropical forage widely used in ruminant production systems, particularly in regions with pronounced dry seasons. With the potential to produce 15 to 20 Mg of total digestible nutrients (TDN) per hectare in a single harvest (Balieiro et al., 2009), sugarcane represents an efficient and economical energy source. Although fresh sugarcane is still utilized in some systems, ensiling has become increasingly important due to its compatibility with mechanized harvesting and its capacity to preserve forage for extended periods.

Despite these advantages, sugarcane silage is highly susceptible to fermentative losses. Its high concentration of water-soluble carbohydrates promotes the growth of epiphytic yeasts that metabolize sugars and organic acids into ethanol and CO₂, resulting in substantial dry matter (DM) and energy losses (Pedroso et al., 2005; Schmidt et al., 2011). These processes reduce silage energy density and palatability, increase fiber concentration, impair digestibility, and ultimately compromise animal performance while increasing feeding costs (Santos et al., 2008; Yang et al., 2025).

A major limitation of conventional silage additives lies in their mode of action. Chemical treatments rely on nonspecific antimicrobial effects that may compromise feed safety or palatability, whereas microbial inoculants depend on competitive metabolic dominance, which is strongly influenced by substrate availability and environmental conditions. Given that gas and effluent losses in sugarcane silage can reach 40–50 % of total DM (Siqueira et al., 2007; Santos et al., 2008; de Sá et al., 2024), these constraints underscore the need for alternative strategies capable of selectively targeting spoilage microorganisms while preserving desirable fermentation processes.

Immunoglobulin Y (IgY) antibodies represent a promising biological alternative. Unlike chemical or microbial additives, IgY acts through specific antigen–antibody interactions, impairing microbial adhesion, colonization, and proliferation. This mechanism may be particularly advantageous in silage systems, as early suppression of spoilage microorganisms under post-harvest pH conditions (5.2–6.8; Misra et al., 2020, 2022) can influence subsequent fermentation dynamics and enhance aerobic stability after silo opening (Bai et al., 2024).

IgY is the principal serum antibody in birds and can be non-invasively harvested from egg yolks, allowing antibody production without the need for mammalian systems (Lee et al., 2021). Unlike mammalian IgG, IgY does not bind to mammalian Fc receptors or activate the mammalian complement cascade, reducing the likelihood of immunogenic or off-target effects when administered orally (Eriksson & Larsson, 2025; Lee et al., 2021; Wang et al., 2023). IgY has been applied in animal health studies to neutralize pathogens and modulate microbial populations without reported adverse effects (Balieiro Neto et al., 2017; Capotă et al., 2025; Karthikeyan et al., 2022; Pacheco et al., 2023), supporting its potential as a targeted biological tool.

This study evaluated a silage additive containing IgY antibodies targeting dominant yeast species associated with sugarcane silage. We hypothesized that anti-yeast IgY suppresses yeast proliferation during the early stages of ensiling, thereby reducing ethanol production and gas losses, improving aerobic stability, and enhancing the overall fermentation quality of sugarcane silage.

## 2 Materials and methods

### 2.1 Ethical approval and yeast strain selection

All experimental procedures involving animals were approved by the Ethics Committee for Animal Use of the Animal Science Institute – IZ/APTA/SAA (Approval No. 262-18). Yeast strains were selected based on their dominance in sugarcane silage and their capacity to ferment sugars and organic acids. Strains included Candida glabrata, Pichia kudriavzevii, Pichia manshurica, Saccharomyces cerevisiae, Schizosaccharomyces pombe, Torulospora delbrueckii, and Debaryomyces etchellsii. All strains were registered in the Brazilian National System for Genetic Resource Management (SISGEN) prior to use.

### 2.2 Ensiling procedures

#### 2.2.1 Forage source and processing

Sugarcane cultivar IAC SP 93-3046 with 28.3° Brix was harvested manually at 18 months of regrowth (second cut). Immediately after harvesting, tops and leaves were removed, and stalks were loaded into a truck lined with a new clean plastic tarp. Chopping was performed using a forage harvester adjusted to 2-cm particle size. This particle size was selected to ensure adequate compaction and anaerobic conditions while maintaining sufficient surface area for microbial activity during fermentation. Chopped cane was placed on tarpaulin-lined ground to prevent contamination.

#### 2.2.2 IgY production and characterization

Yeast antigens were prepared from strains belonging to the Culture Collection of Agricultural Microbiology (CCMA) of the Department of Biology at the Federal University of Lavras (UFLA-MG) and from the Institute of Biological Sciences of the Federal University of Minas Gerais (UFMG). The following strains were used: *Candida glabrata* (CCMA 0486), isolated from sugarcane silage; *Pichia kudriavzevii* (CCMA 0051), isolated from coffee fermentation; *Pichia manshurica* (CCMA 0048), isolated from sugarcane silage; *Saccharomyces cerevisiae* (CCMA 0491), isolated from apple; *Torulospora delbrueckii* (CCMA 0470), isolated from coffee; *Schizosaccharomyces pombe* (CCMA 0050), isolated from sugarcane silage and *Debaryomyces etchellsii* (CCMA 0045), isolated from sugarcane. These strains were selected based on their documented occurrence in sugarcane environments and silage systems, as well as their metabolic capacity to ferment sugars and organic acids, characteristics directly associated with fermentative losses in sugarcane silage. Strains are maintained in the laboratory collections and are available for replication purposes. Yeasts were cultivated under laboratory conditions, harvested by centrifugation, washed in phosphate-buffered saline (PBS), chemically inactivated with formaldehyde, and combined into a single antigen suspension formulated with aluminum hydroxide adjuvant.

For antibody production, 25-week-old White Leghorn laying hens were immunized via intramuscular injection in the pectoral muscle with 0.5 mL of the antigen–adjuvant suspension. A control group received adjuvant plus PBS only. Eggs were collected during the production period, and yolks were manually separated, freeze-dried, and delipidated. IgY was extracted from the lyophilized yolk using polyethylene glycol precipitation and resuspended in PBS.

The lyophilized product presented 96.46 % dry matter, 38.98 % crude protein, 46.01 % ether extract, 0.59 % crude fiber, 4.78 % mineral matter, 9.65 % nitrogen-free extract, and 130.69 % total digestible nutrients (on a dry matter basis). IgY concentration was determined using a commercial Chicken IgY ELISA kit (Innovative Research Inc.), with absorbance measured at 450 nm. The calibration curve showed high goodness-of-fit (R² = 0.985), and the final IgY concentration in the product was 11.5 mg g⁻¹. Antibody bioactivity was confirmed by Plate-Trapped Antigen (PTA)-ELISA, demonstrating specific antigen–antibody complex formation against the targeted yeast strains, thereby confirming recognition capacity and functional activity of the IgY preparation.

#### 2.2.3 Treatments

A lyophilized IgY-based silage additive containing specific antibodies against the yeast species *Candida glabrata, Pichia kudriavzevii, Pichia manshurica, Schizosaccharomyces pombe, Saccharomyces cerevisiae, Torulaspora delbrueckii*, and *Debaryomyces etchellsii* was used. The additive was homogenized with chopped sugarcane immediately before ensiling to ensure uniform distribution at rates of 0, 175, 350, and 700 g/t of IgY of fresh forage. The control treatment (0 g/t) received a lyophilized product derived from hens inoculated with adjuvant only (without yeast antigens), thus devoid of anti-yeast antibodies, allowing isolation of the antibody effect.

An additional treatment consisted of ensiling sugarcane with a microbial inoculant containing *Lactobacillus buchneri*. The inoculant was applied at 2 g t⁻¹ of commercial product, providing 5.0 × 10¹⁰ CFU t⁻¹ of fresh forage. The inoculant was diluted in deionized water and uniformly sprayed onto the chopped forage, according to the manufacturer’s instructions.

#### 2.2.4 Experimental silos and ensiling

Experimental silos consisted of 20-L plastic buckets equipped with Bunsen valve lids to allow gas release. Each silo was loaded with 3.5 kg of sand, separated from the forage mass by a plastic mesh, to enable effluent collection. For each replicate, fresh chopped sugarcane was independently weighed and treated with the respective additive dose, thoroughly homogenized, and ensiled into an individual silo.

Initially, a single silo was filled, compacted using a cement tamper, and weighed to determine the target specific mass (750 kg m⁻³). Based on this initial standardization, the same fresh forage mass was weighed and applied to all subsequent silos prior to ensiling, ensuring uniform specific mass across experimental units. This procedure guaranteed consistent compaction and minimized oxygen entrapment, thereby standardizing fermentation dynamics among treatments under conditions representative of practical farm-scale ensiling.

Silos were sealed with lids and adhesive tape and stored under shelter for 114 days, a period sufficient to complete the fermentation process and simulate typical storage durations across production seasons. A completely randomized design with six treatments and five replicates was adopted.

#### 2.2.5 Sampling and chemical analyses

Samples were collected at ensiling and upon silo opening. For dry matter (DM) determination, samples were dried at 65 °C for 72 h in a forced-air oven. Ground samples were analyzed for crude protein (CP), ether extract (EE), and ash according to Silva & Queiroz (2002). Neutral detergent fiber (NDF) and acid detergent fiber (ADF) were determined as per Van Soest et al. (1991). Non-fiber carbohydrates (NFC) and total carbohydrates (CHO) were estimated using NFC = 100 - (NDF + CP + EE + Ash) and CHO = 100 - (CP + EE + Ash), respectively.

#### 2.2.6 Fermentation losses

Total DM loss (DML) was calculated as the difference between initial and final DM (Jobim et al., 2007) by DML = ((FMs – FMo) / FMs) × 100, where: FMs = forage mass at sealing, FMo = forage mass at opening. Gas losses (G) were calculated based on weight differences of silos pre- and post-storage using the formula G = (SWs – SWo) / FMs × 100, where: SWs = silo weight at sealing, SWo = silo weight at opening, and FMs = forage mass at sealing. Effluent production (E) was estimated using the formula E (kg/t) = ((Wo – We) × 100) / FFe, where: Wo = weight of the bucket + sand + mesh at opening (kg), We = weight of the bucket + sand + mesh at ensiling (kg) and FFe = fresh forage mass ensiled (kg).

#### 2.2.7 Silage quality parameters

At silo opening, after 114 days of ensiling, the entire contents of each silo were thoroughly homogenized. A 200 g subsample was subjected to hydraulic pressing (2 kgf/cm²) to obtain silage juice for subsequent chemical analyses. Lactic acid concentrations were determined by high-performance liquid chromatography (HPLC), following the methodology described by Zhao et al. (2022). Volatile fatty acids (acetic, propionic, and butyric acids), as well as ethanol, were quantified by gas chromatography, as outlined by Deeken et al. (2024). Ammoniacal nitrogen (N‑NH₃) was measured using a colorimetric method (Zhao et al., 2022). Soluble sugar content (°Brix) was assessed with a handheld refractometer. Silage pH was determined by homogenizing 25 g of silage in 225 mL of deionized water for 1 minute in a blender, with measurements performed using a benchtop pH meter, according to Cabrera‑Ortiz et al. (2019).

#### 2.2.8 Yeast population analysis

Yeast populations were quantified by homogenizing 25 g of silage in 225 mL of sterile saline solution (8.5 g NaCl/L), followed by shaking at 30 °C for 4 minutes. Samples were serially diluted (10⁻², 10⁻³, and 10⁻⁴) and aliquots were plated on 3M™ Petrifilm™ Yeast and Mold Count Plates, according to the methodology described by Schumacher et al. (2023). The plates were incubated at 30 °C for 48 hours. Colony counts were performed at 0, 24, 48, and 72 hours after exposure of the silage to aerobic conditions. Plates showing no visible yeast colonies were recorded as below the detection limit of the method (LOD < 100 CFU g⁻¹). The LOD was calculated considering 1 CFU per plate as the minimum detectable level, corresponding to the plating of 1 mL of the 10⁻² dilution. Yeast counts were expressed as log CFU g⁻¹ of fresh matter.

#### 2.2.9 Aerobic stability assessment

Aerobic stability was monitored for 3 days post-opening by recording silage temperature every 5 min using thermocouples placed in 4 kg of homogenized silage in plastic buckets. A second bucket was used to measure daily pH and DM. Aerobic stability parameters followed Del Valle et al. (2022), including time to 2 °C rise (*h T>2* *°C*), time to peak temperature (*h T max*) and maximum temperature (*T max*). Aerobic stability parameters such as time to 2 °C temperature rise (*h T>2* *°C*) are increasingly used in tropical silage research to quantify aerobic deterioration (Monção et al., 2024).

#### 2.2.10 Dry matter recovery (DMR)

DMR during storage was calculated following Bernardes et al. (2007): DMR (%) = (MFf × MSf) / (MFi × MSi) × 100, where: MFf = final forage mass, MSf = final DM content, MFi = initial forage mass and MSi = initial DM content.

#### 2.2.11 Statistical analysis

Data were analyzed using two complementary approaches to ensure both treatment comparison and dose–response evaluation. For overall treatment effects, all six treatments (Control, *Lactobacillus buchneri*, and IgY at 0, 175, 350, and 700 g t⁻¹) were analyzed using one-way analysis of variance (ANOVA) under a completely randomized design. When significant effects were detected (*P* < 0.05), means were separated using Tukey’s multiple comparison test at the 5 % significance level.

To evaluate dose–response relationships among IgY inclusion levels, regression analyses were performed considering IgY dose (0, 175, 350, and 700 g t⁻¹) as a quantitative independent variable. Additionally, orthogonal polynomial contrasts (linear and quadratic) were applied to formally test dose–response trends across IgY inclusion levels. Linear and quadratic models were tested for selected fermentation parameters (acetic acid, lactic acid and ethanol concentrations), as well as for pH, dry matter recovery after five days of aerobic exposure, and gas losses. The best-fitting model was selected based on significance of regression coefficients and biological interpretability. Model assumptions were evaluated through residual diagnostics, including tests for normality (Shapiro–Wilk) and visual inspection for homoscedasticity. Ninety-five percent confidence intervals were calculated for predicted ethanol means to support model interpretation.

Yeast counts were log₁₀-transformed prior to analysis to meet normality assumptions. In samples where no colony growth was detected on 3M™ Petrifilm™ Yeast and Mold Count Plates, values were assigned the detection limit of the method prior to log transformation to allow statistical inclusion. All statistical analyses were performed using SAS software (SAS Institute Inc., 2013, Cary, NC, USA; version 9.4).

## 3 Results

Brix values in silage juice were lower (*P* < 0.05) in the control and in silages treated with 0 and 175 g t⁻¹ of IgY compared with the other treatments, whereas no differences were observed among silages treated with 350 and 700 g t⁻¹ of IgY and *Lactobacillus buchneri*. Silages treated with 0 and 175 g t⁻¹ IgY did not reduce yeast populations effectively (*P* > 0.05), thus these treatments were included for completeness but did not significantly impact fermentation or yeast control (Table 1).

**Table 1:** Silage juice Brix and yeast populations (0 and 24 h) of sugarcane silages treated with Lactobacillus buchneri or supplemented with yeast-targeting IgY antibodies.

			IgY (g t^−1^)					
Parameter	Control	*L. buchneri*	0	175	350	700	SEM	*P* – value
Silage juice Brix	15.53 ^b^	20.70 ^a^	18.43 ^b^	17.45 ^b^	23.70 ^a^	24.26 ^a^	1.885	<0.001
Yeast (0h), log CFU g^−1^	4.56 ^a^	1.65 ^b^	5.87 ^a^	4.46 ^a^	LOD	LOD	1.246	0.014
Yeast (24h), log CFU g^−1^	4.87	4.04	5.87	4.36	LOD	LOD	1.232	0.348

Different letters within a row indicate significant differences (P < 0.05). LOD = yeast counts below the detection limit of the method (< 100 CFU g⁻¹), determined based on plating 1 mL of the 10⁻² dilution on 3M™ Petrifilm™ Yeast and Mold Count Plates. For statistical analysis, LOD values were assigned the numerical value of the detection limit (100 CFU g⁻¹) prior to log₁₀ transformation.

Yeast counts at silo opening (0 h) were reduced (*P* < 0.05) in the *L. buchneri* treatment compared with the control. In contrast, silages treated with IgY at doses ≥ 350 g t⁻¹ showed yeast counts below the detection limit of the method (<100 CFU g⁻¹) at silo opening and after 24 h of aerobic exposure (Table 1).

Silage pH differed among treatments throughout the aerobic exposure period (Table 2). At silo opening (0 h), silages supplemented with IgY exhibited lower pH values than the control, with the lowest pH observed in the silage treated with 350 g t⁻¹ IgY (*P* < 0.05). After 24 h of aerobic exposure, the control silage showed the highest pH among treatments, whereas IgY-treated silages maintained lower pH values. At 48 h after silo opening, pH remained higher in the control silage compared with the other treatments. After 72 h of aerobic exposure, only the silage treated with 350 g t⁻¹ IgY maintained a pH value lower than that of the control silage (Table 2).

**Table 2:** pH values of sugarcane silages treated with Lactobacillus buchneri or supplemented with yeast-targeting IgY antibodies at different timepoints after silo opening.

			IgY level (g/t)					
Hours after opening	Control	*L. buchneri*	0	175	350	700	SEM	*P* – value
0	2.64 ^bc^	2.53 ^c^	2.88 ^a^	2.72 ^b^	2.36 ^d^	2.55 ^c^	0.091	0.0001
24	3.31 ^a^	3.03 ^b^	2.90 ^c^	2.82 ^c^	2.88 ^c^	3.05 ^b^	0.134	0.0008
48	2.72 ^b^	2.51 ^c^	2.91 ^a^	2.87 ^ab^	2.33 ^c^	2.48 ^c^	0.129	0.0005
72	3.13 ^a^	2.90 ^b^	2.93 ^b^	2.71 ^d^	2.83 ^c^	2.89 ^b^	0.085	0.0007

Different superscript letters within a row indicate significant differences (P < 0.05).

**Fig. 1:**
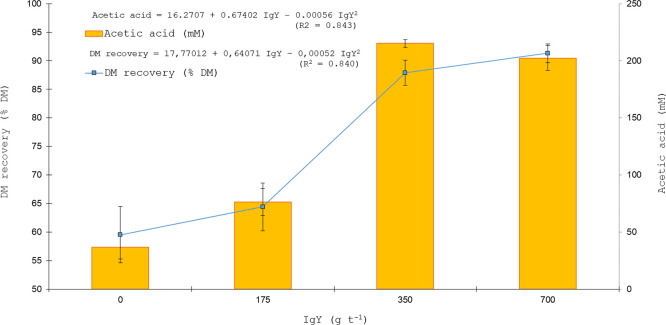
Quadratic regression analysis of DM recovery (% DM) after five days of aerobic exposure and acetic acid concentration (mM) in sugarcane silage as affected by IgY inclusion levels (0, 175, 350, 700 g t⁻¹).

**Fig. 2:**
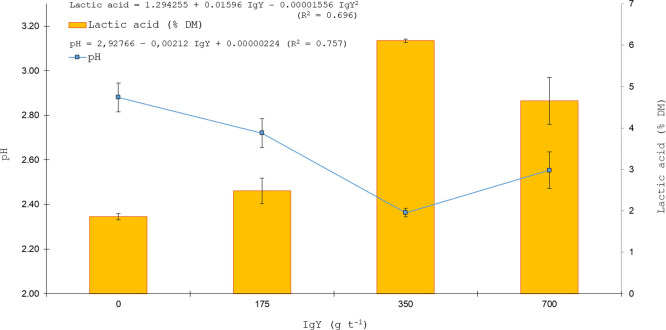
Linear regression analysis of pH and quadratic regression of lactic acid concentration (% DM) in sugarcane silage as affected by IgY inclusion levels (0, 175, 350, 700 g t⁻¹).

**Fig. 3:**
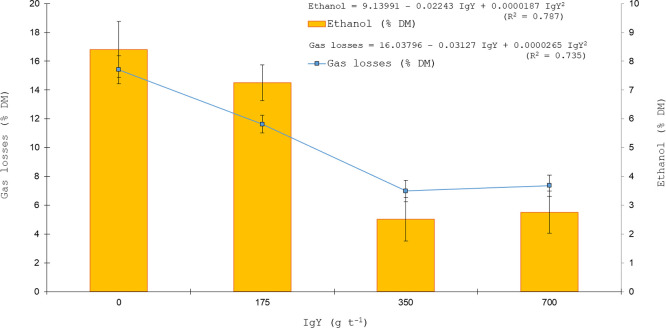
Quadratic regression analysis of ethanol concentration (% DM) and gas losses (% DM) in sugarcane silage as affected by IgY inclusion levels (0, 175, 350, 700 g t⁻¹).

The organic acid profile of sugarcane silages was influenced by IgY inclusion level, as demonstrated by quadratic regression analyses (Fig. 1, Fig. 2, Fig. 3). Fig. 1 presents the quadratic regression of acetic acid concentration along with DM recovery after five days of aerobic exposure, whereas Fig. 2 illustrates the quadratic regression of lactic acid concentration in association with pH values, and Fig. 3 shows the quadratic regression of ethanol concentration together with gas losses.

Quadratic regression analyses were used to characterize the dose–response relationships between IgY supplementation and key fermentation variables. The fitted models showed substantial explanatory power, with coefficients of determination (R²) of 0.843 for acetic acid (*P* < 0.0001), 0.840 for dry matter recovery after aerobic exposure (*P* = 0.0001), 0.697 for lactic acid (*P* = 0.0026), 0.802 for pH (*P* = 0.0003), 0.788 for ethanol (*P* < 0.0001), and 0.736 for gas losses (*P* = 0.0013). These statistics indicate that the quadratic models adequately captured the nonlinear responses of fermentation products, pH dynamics, and dry matter losses to increasing IgY inclusion.

Acetic and lactic acid concentrations were higher in silages treated with IgY at ≥ 350 g t⁻¹, consistent with reduced yeast activity, whereas ethanol concentrations were correspondingly lower (Fig. 1, Fig. 2, Fig. 3). Residual diagnostics confirmed the adequacy of the quadratic regression models for acetic acid, lactic acid, and ethanol. Normality of residuals was verified using the Shapiro–Wilk test, with *P* = 0.80 for acetic acid, *P* = 0.68 for lactic acid, and *P* = 0.73 for ethanol, indicating no significant deviation from normality. No evidence of heteroscedasticity was observed for any of the models. Furthermore, 95 % confidence intervals were calculated for the predicted means of each variable, supporting the robustness and reliability of the fitted models. These diagnostic results reinforce the validity of the observed dose–response relationships and justify the use of the quadratic regressions for interpreting treatment effects. Orthogonal polynomial contrasts were performed to formally test the dose–response patterns observed in the regression analyses (Fig. 1, Fig. 2, Fig. 3). Acetic and lactic acid concentrations exhibited significant linear and quadratic effects of increasing IgY dose (acetic acid: linear *P* < 0.0001, quadratic *P* = 0.0023; lactic acid: linear *P* < 0.0001, quadratic *P* = 0.0010), whereas ethanol concentration showed a significant linear decrease with increasing IgY dose (linear *P* < 0.0001; quadratic *P* = 0.2057).

The concentrations of organic acids, ammonia nitrogen, lactic acid, and ethanol were affected by both *L. buchneri* inoculation and IgY supplementation (Table 3). Acetic acid concentration was lowest in the control silage, increased in *L. buchneri*–treated silage, and was greater in silages supplemented with IgY at 350 and 700 g t⁻¹. Propionic acid concentration was also higher in IgY-treated silages, with the highest value observed at 700 g t⁻¹. In contrast, butyric and valeric acid concentrations were greatest in the control silage and were reduced in both *L. buchneri*–treated and IgY-treated silages. Isobutyric and isovaleric acid concentrations did not differ among treatments. Ammonia nitrogen concentration was greater in IgY-treated silages than in the control and *L. buchneri* treatments, with the highest values observed at 700 g t⁻¹. Lactic acid concentration was greater in IgY-treated silages, particularly at 350 g t⁻¹, compared with the control and *L. buchneri* treatments. Conversely, ethanol concentration was highest in the control silage and was reduced in both *L. buchneri*–treated and IgY-treated silages, with the lowest value observed at 350 g t⁻¹ of IgY (Table 3).

**Table 3:** Organic acid profiles, ammonia nitrogen, lactic acid, and ethanol concentrations in sugarcane silages treated with Lactobacillus buchneri or supplemented with yeast-targeting IgY antibodies.

			IgY (g t^−1^)					
Parameter	Control	*L. buchneri*	0	175	350	700	SEM	*P* – value
Acetic acid (mM)	58.65 ^e^	152.44 ^c^	36.48 ^f^	76.36 ^d^	212.74 ^a^	202.35 ^b^	10.07	<0.0001
Propionic acid (mM)	0.00 ^d^	1.34 ^bc^	0.92 ^bc^	1.31 ^bc^	1.39 ^ab^	1.96 ^a^	0.34	0.0002
Butyric acid (mM)	0.79 ^a^	0.36 ^c^	0.45 ^b^	0.37 ^c^	0.23 ^d^	0.39 ^c^	0.20	0.008
Isobutyric acid (mM)	0.72 ^bc^	0.68 ^d^	0.66 ^d^	0.69 ^cd^	0.74 ^ab^	0.77 ^a^	0.06	0.0018
Valeric acid (mM)	3.14 ^a^	1.79 ^b^	0.78 ^c^	0.84 ^c^	0.90 ^c^	1.39 ^bc^	0.44	<0.0001
Isovaleric acid (mM)	0.00	0.05	0.00	0.00	0.00	0.00	0.29	0.322
Ammonia-N (mg/dL)	1.35 ^c^	2.01 ^c^	6.26 ^b^	7.46 ^b^	8.26 ^b^	15.54 ^a^	1.77	<0.0001
Lactic acid (g/kg DM)	16.0 ^d^	21.3 ^cd^	18.6 ^d^	24.8 ^c^	61.0 ^a^	46.5 ^b^	3.88	<0.0001
Ethanol (g/kg DM)	82.7 ^a^	38.9 ^b^	84.1 ^a^	72.4 ^a^	25.1 ^c^	27.5 ^bc^	8.13	<0.0001

Different superscript letters within a row indicate significant differences (P < 0.05).

An increase of 2 °C above ambient temperature (h T > 2 °C) was observed in the control and *Lactobacillus buchneri*-treated silages (Table 4). Notably, silages treated with 350 and 700 g t⁻¹ of the IgY-based additive did not exceed this threshold during the monitoring period. The control silage reached its thermal peak 84 h after air exposure, whereas silages treated with 350 and 700 g t⁻¹ IgY reached their peaks earlier, at 21 h and 1.72 h, respectively. Maximum temperatures (T max) did not differ significantly among treatments.

**Table 4:** Temperature dynamics and aerobic stability in sugarcane silages treated with Lactobacillus buchneri or supplemented with yeast-targeting IgY antibodies.

			IgY (g t^−1^)					
Parameter (%)	Control	*L. buchneri*	0	175	350	700	SEM	*P* – value
T max (C^o^)	27.45 ^ab^	26.53 ^b^	27.82 ^ab^	30.86 ^a^	26.05 ^b^	25.92 ^b^	2.547	0.231
h T max (h)	84 ^bc^	111^ab^	57 ^dc^	132^a^	20.53^de^	1.72^e^	27.884	0.0003
h T > 2°C (h)	4.61 ^c^	66.36 ^b^	3.48 ^c^	78.1 ^a^	ND	ND	3.253	<0.0001

T max = maximum temperature reached by the silage after air exposure; h T max = time (h) for the silage to reach the T max; h T >2 °C = time (h) for an increase of 2 °C above ambient temperature. Means within a row followed by different letters differ (P < 0.05). ND = not detected; h T > 2 °C was not reached in silages treated with 350 and 700 g t⁻¹ IgY, and therefore statistical analysis was not applicable.

Fermentative losses and dry matter (DM) recovery are presented in Table 5. Dry matter losses were greater (*P* < 0.05) in the control silage than in the treated silages, with no differences observed among *L. buchneri* and IgY treatments. Gas losses were reduced (*P* < 0.05) in silages treated with IgY at doses ≥ 350 g t⁻¹ and were also lower in the *L. buchneri* treatment compared with the control. Effluent losses were not significantly affected by treatments (*P* = 0.055), although a tendency for lower values was observed in silages supplemented with IgY. Dry matter recovery during fermentation was higher in silages treated with IgY at 350 and 700 g t⁻¹ (87.9 % and 91.3 %, respectively) compared with the control and *L. buchneri*-treated silages (Table 5). After five days of aerobic exposure, dry matter recovery differed among treatments, with the lowest value observed in the control silage (67.7 %). Silages treated with IgY at 350 and 700 g t⁻¹ maintained greater dry matter recovery after aerobic exposure (87.5 % and 85.2 %, respectively) compared with the control and were numerically higher than the *L. buchneri* treatment (Table 5).

**Table 5:** Fermentative losses, chemical characteristics, and yeast populations in sugarcane silages treated with Lactobacillus buchneri or supplemented with yeast-targeting IgY antibodies.

			IgY (g t^−1^)					
Parameter	Control	*L. buchneri*	0	175	350	700	SEM	*P* – value
Initial DM (%)	36.47 ^b^	36.81 ^b^	36.49 ^b^	37.07 ^ab^	36.75 ^b^	37.68 ^a^	0.361	0.003
Final DM (%)	28.14 ^b^	32.90 ^ab^	35.66 ^a^	36.50 ^a^	35.23 ^a^	35.32 ^a^	1.854	<0.001
DM losses (% DM)	32.26 ^a^	19.68 ^ab^	14.02 ^b^	11.70 ^b^	12.47 ^b^	14.80 ^b^	4.950	<0.001
DM recovery* (% DM)	67.73 ^b^	80.31 ^ab^	85.98 ^a^	88.29 ^a^	87.52 ^a^	85.20 ^a^	4.950	<0.001
DM recovery** (% DM)	81.72 ^a^	85.61 ^a^	59.53 ^b^	64.42 ^b^	87.87 ^a^	91.31^a^	5.328	<0.0001
Gas losses (% DM)	16.21 ^a^	10.10 ^b^	15.36^a^	12.72 ^ab^	6.99 ^c^	7.35 ^c^	2.194	<0.001
Effluent losses (kg t^−1^)	64.06	64.42	63.08	56.06	56.74	60.15	4.087	0.055

Means within a row followed by different letters differ at P < 0.05.

DM recovery* = DM recovery during the fermentation phase.

DM recovery** = DM recovery after five days of aerobic exposure following silo opening.

The chemical composition of sugarcane silages differed among treatments (Table 6). Dry matter content was greater in silages supplemented with IgY at 350 and 700 g t⁻¹ compared with the control, while *L. buchneri*-treated silage showed intermediate values. Crude protein and ether extract increased progressively with IgY inclusion, reaching the highest values at 700 g t⁻¹. Structural carbohydrate fractions (ADF, NDF, hemicellulose, and cellulose) were highest in the control, lower in IgY-treated silages, and intermediate in *L. buchneri*-treated silage. Lignin was reduced in silages treated with 700 g t⁻¹ IgY. NFC concentration was highest in *L. buchneri*-treated silage, intermediate in IgY 350 g t⁻¹, and lowest in the control and IgY 700 g t⁻¹. Ash differed slightly among treatments, with the control highest, *L. buchneri* lowest, and IgY-treated silages intermediate.

**Table 6:** Chemical composition (%) of sugarcane silages treated with *Lactobacillus buchneri* or supplemented with yeast-targeting IgY antibodies.

			IgY (g t^−1^)					
Parameter (%)	Control	*L. buchneri*	0	175	350	700	SEM	*P* – value
Dry matter	26.50 ^b^	30.89 ^a^	32.30 ^a^	32.88 ^a^	32.63 ^a^	32.95^a^	1.81	0.005
Crude protein	2.16 ^e^	1.76 ^f^	6.61 ^b^	5.33 ^c^	4.77 ^d^	7.20 ^a^	0.26	<.0001
Ether extract	1.58 ^e^	1.45 ^e^	3.69 ^c^	3.26 ^d^	6.51 ^b^	9.90 ^a^	0.27	<.0001
ADF	34.94 ^a^	29.17 ^b^	35.05 ^a^	30.37 ^b^	28.61 ^bc^	25.68 ^c^	2.21	0.0009
NDF	53.33 ^a^	44.96 ^b^	53.57 ^a^	46.67 ^b^	43.68 ^bc^	40.07 ^c^	2.93	0.0012
Hemicellulose	18.38 ^a^	15.79 ^bc^	18.51 ^a^	16.30 ^b^	15.07 ^bc^	14.39 ^c^	1.18	0.0036
Cellulose	29.02 ^a^	23.88 ^bc^	29.21 ^a^	25.20 ^b^	23.29 ^bc^	21.05 ^c^	2.21	0.003
Lignin	5.29 ^a^	5.49 ^a^	5.25 ^a^	4.59 ^b^	4.58 ^b^	4.30 ^b^	0.33	0.002
NFC	41.02 ^b^	50.32 ^a^	34.33 ^c^	42.85 ^b^	43.36 ^b^	41.28 ^b^	3.58	0.003
Ash	1.89	1.49	1.79	1.87	1.66	1.53	0.24	0.232

Different letters within a row indicate significant differences (P < 0.05).

## 4 Discussion

At harvest, sugarcane juice typically exhibits a pH ranging from 5.2 to 5.8, depending on cultivar and field conditions (Misra et al., 2022), with post-harvest values reported as high as 6.8 (Misra et al., 2020). These moderately acidic and sugar-rich conditions are highly favorable for yeast proliferation. The IgY application during chopping or immediately prior to ensiling suppressed yeast populations before extensive lactic acid accumulation and pH decline occurred. This early inhibition is consistent with the complete absence of detectable yeast growth in silages treated with IgY at inclusion levels ≥ 350 g t⁻¹ at silo opening and during subsequent aerobic exposure. In contrast, silages treated with 0 and 175 g t⁻¹ IgY did not show a reduction in yeast populations compared with the control, confirming that these lower doses were biologically ineffective in controlling yeast growth and had limited influence on fermentation outcomes. By limiting yeast proliferation during the initial phase of fermentation, IgY-treated silages reduced subsequent lactic acid degradation and sugar consumption, thereby retaining greater amounts of fermentable substrates and exhibiting a more favorable fermentation profile throughout storage.

Although quadratic responses were observed for acetic acid, lactic acid, ammonia nitrogen, and ethanol, the regression models were fitted using only four dose levels (0, 175, 350, and 700 g/t). This limited the residual degrees of freedom, therefore, these dose-response relationships should be interpreted cautiously.

The initial suppression of yeast activity in IgY-treated silages resulted in lower yeast populations after silo opening, thereby favoring aerobic stability, particularly at inclusion levels ≥ 350 g t⁻¹. This response is consistent with recent evidence suggesting that the conventional 72-h criterion for aerobic stability may underestimate stability improvements in tropical silages (de Sá et al., 2024). The lower yeast populations observed after silo opening in the present study reflect the antimicrobial efficacy of IgY in limiting yeast growth and metabolic activity during the initial phase of fermentation, prior to the decline in pH, which in turn reduced the subsequent consumption of organic acids with preservative and antifungal properties, thereby delaying silage deterioration upon air exposure. Although the 0 and 175 g t⁻¹ treatments were included in the tables for completeness, they did not meaningfully affect yeast counts, pH, or dry matter preservation and were therefore considered ineffective doses for practical application.

In the context of silage production, IgY is not intended to function as a therapeutic antibody but rather as a locally acting biological inhibitor with a limited functional window. Its primary activity occurs during the early phase of ensiling, when pH values remain relatively high and yeast populations are most active. Even partial antibody binding during this period may be sufficient to alter yeast population dynamics, resulting in sustained effects on fermentation outcomes. This interpretation is supported by recent reviews highlighting the binding capacity and functional stability of IgY antibodies in food and feed systems (Capotă et al., 2025).

Concerns regarding potential IgY denaturation under acidic conditions largely stem from ex vivo digestive models, such as the chicken gizzard, where degradation occurs rapidly due to the combined effects of low pH and digestive enzymes. However, exposure to acidic conditions alone does not appear to compromise IgY integrity, as demonstrated under controlled conditions at pH 3 (Wang et al., 2021). This relative acid tolerance is attributed to structural features of IgY, including an additional CH4 domain, which confers greater stability than mammalian IgG across a range of environmental stressors (Gandhi & Alshehri, 2021).

The IgY preparation used in the present study was designed to specifically target yeasts, and its mode of action is biological rather than metabolic. IgY antibodies bind to yeast cell-surface antigens, promoting cell agglutination, impairing nutrient uptake, and reducing metabolic activity or viability. Consequently, suppression of yeast populations limits lactic acid utilization after fermentation and during storage, allowing greater retention of this organic acid. The increased lactic acid concentrations observed in IgY-treated silages are therefore more plausibly attributed to reduced degradation by yeasts than to a direct stimulation of lactic acid bacteria growth.

The elevated ammonia-N concentrations observed in silages treated with the 0, 175, 350 and 700 g t⁻¹ treatments are largely attributable to the protein content of the IgY additive itself, which is delivered in an egg-yolk matrix. Proteolytic degradation of this protein-based additive during the 114-day ensiling period contributes to the increased ammonia-N, rather than reflecting excessive degradation of the forage protein. Furthermore, no parallel increase in butyric acid was observed, indicating that the elevated ammonia-N was not associated with clostridial activity.

Increased ammonia‑N in IgY‑supplemented silages likely reflects a combination of rapid deamination of highly soluble egg yolk protein and enhanced bacterial turnover due to suppression of yeasts, which alters microbial competition and substrate availability (Sun et al., 2021; Tuovinen et al., 2025). Some naturally occurring lactic acid bacteria (e.g., *Lactiplantibacillus plantarum, Pediococcus pentosaceus, Enterococcus faecium*) and early‑colonizing enterobacteria (*Enterobacter cloacae, Klebsiella pneumoniae*) possess proteolytic activity that can increase ammonia formation during ensiling (Der Bedrosian et al., 2012; Kleinschmit & Kung, 2006). Therefore, the observed increase in ammonia‑N at higher IgY doses is consistent with a synergistic effect of the degradable protein vehicle and shifts in microbial metabolism during fermentation.

Consistent with this mechanism, IgY-treated silages exhibited lower ethanol concentrations, reinforcing the role of yeast inhibition in minimizing energy losses during storage. The concomitant reduction in gas losses associated with decreasing ethanol concentrations (Fig. 3) further supports the interpretation that yeast suppression directly limited fermentative carbon losses. Ethanol production in sugarcane silage is predominantly associated with fermentative yeasts, which readily proliferate in sugar-rich substrates. Effective suppression of these microorganisms by IgY antibodies therefore directly limits ethanol formation, contributing to reduced dry matter losses and improved preservation efficiency.

Acetic acid plays a critical antifungal role in silage preservation during aerobic exposure by inhibiting yeasts and molds, the primary agents of aerobic spoilage. Although *Lactobacillus buchneri* is widely used to enhance acetic acid production through heterofermentative pathways, the higher acetic acid concentrations observed in IgY-treated silages suggest a distinct mechanism. In this case, increased acetic acid accumulation appears to result primarily from reduced microbial utilization, particularly by yeasts, rather than from enhanced heterofermentative activity. Thus, the higher acetic acid concentrations likely reflect improved retention rather than enhanced heterofermentative conversion.

The elevated acetic acid concentrations observed in silages treated with *L. buchneri* and, more notably, in those supplemented with IgY at 350 and 700 g t⁻¹ are indicative of enhanced antifungal potential and improved aerobic stability. While the response observed with *L. buchneri* is consistent with its established metabolic pathway involving lactic acid conversion to acetic acid, the response in IgY-treated silages reflects suppression of spoilage microorganisms rather than altered bacterial metabolism.

The control silage exhibited higher neutral detergent fiber (NDF) and acid detergent fiber (ADF) contents compared with silages treated with active IgY antibodies, indicating that IgY itself played a critical role in preserving soluble carbohydrates during fermentation. The reduced fiber fractions (NDF, ADF, cellulose, and hemicellulose) observed in IgY-treated silages likely reflect improved preservation of soluble components, associated with more effective control of spoilage microorganisms, particularly yeasts (Araújo et al., 2023). Considering the negative relationship between fiber content and silage intake and digestibility, these findings further support the potential of IgY-based additives to enhance the nutritional quality of sugarcane silages.

In the control treatment, pH was already below 3.5 at 0 h (2.64) and increased to 3.13 at 72 h, indicating that the silages remained within the range considered indicative of adequate fermentation. The quadratic association between lactic acid concentration and pH observed across IgY inclusion levels (Fig. 2) reinforces the central role of organic acid accumulation in modulating the acidification pattern of the silages. This slight decline in pH between 24 and 48 h after silo opening may reflect residual fermentation activity and continued organic acid production, particularly in silages where yeast growth was effectively suppressed, thereby limiting acid consumption during aerobic exposure. In sugarcane silages, the high concentration of soluble carbohydrates promotes rapid fermentation, which generally results in lower final pH values than those observed in other forages, with values close to or below 3.5 being indicative of efficient fermentation and effective preservation of the ensiled mass (Rodrigues et al., 2020). However, yeasts are able to survive and proliferate under low pH conditions, including values below 3.5, which may contribute to silage deterioration even when pH remains within the range considered indicative of good fermentative quality (Rodrigues et al., 2020).

Although an increase in temperature typically indicates microbial activity during aerobic exposure, interpretation should consider both the magnitude of the peak temperature (T max) and whether the increase persists or is interrupted over time. In the present study, the early thermal peaks observed in IgY-treated silages (20.52 h for 350 g t⁻¹; 1.72 h for 700 g t⁻¹) do not correspond to microbial heating but likely reflect minor physical fluctuations upon silo opening or transient thermocouple responses. Yeast-mediated temperature increases require a longer lag phase. Despite reaching an early T max, the temperature increase remained below the 2 °C threshold, characterizing physical fluctuation rather than biological heating. Indeed, these silages never exceeded the 2 °C threshold above ambient (h T > 2 °C = ND), confirming their aerobic stability. In comparison, *L. buchneri*-treated silages reached the thermal peak at 111.28 h, later than the control (84 h), consistent with improved aerobic stability due to heterofermentative acetic acid production.

Well‑preserved silages generally show minimal and transient temperature rises because rapid acidification, accumulation of organic acids, and suppression of spoilage organisms limit aerobic deterioration. In sugarcane silages, higher concentrations of acetic acid have been associated with improved aerobic stability and reduced temperature increases following silo opening, reflecting effective control of yeast and spoilage microorganisms during air exposure (Carvalho et al., 2015). Consequently, silages with higher lactic acid retention and an optimal balance between lactic and acetic acids pose a greater challenge for microbial spoilage after opening. In the present study, while maximum temperatures did not differ significantly among treatments, the control and *L. buchneri*‑treated silages exhibited prolonged progressive temperature increases, whereas silages treated IgY (with 350 and 700 g t⁻¹) reached thermal peaks earlier and did not continue to heat, consistent with reduced yeast activity and higher acetic acid levels in those silages.

Although IgY does not directly stimulate lactic acid bacteria, its indirect effects through yeast suppression are consistent with previous studies demonstrating that inhibition of yeast growth results in greater lactic acid retention and improved fermentation stability. Similar responses have been reported in sugarcane silages treated with *L. buchneri*, highlighting the importance of microbial modulation in fermentation quality (Del Valle et al., 2022). The elevated acetic acid concentrations observed in IgY-treated silages further support the role of early yeast suppression in enhancing aerobic stability, as reported for tropical silages under controlled microbial conditions (Coutinho et al., 2020).

The improved dry matter recovery observed in silages treated with *L. buchneri* and IgY at 350 and 700 g t⁻¹ further confirms enhanced aerobic stability. The quadratic relationship between acetic acid concentration and DM recovery after five days of aerobic exposure (Fig. 1) indicates that enhanced retention of antifungal organic acids contributed to the reduction of post-opening dry matter losses. The remarkable increases in crude protein and ether extract observed in IgY-treated silages largely reflect the protein- and lipid-rich composition of the egg yolk used as the IgY vehicle, in addition to the concentration effect associated with improved dry matter preservation. In contrast, the 0 and 175 g t⁻¹ IgY treatments (egg yolk without active or sufficient antibodies) did not inhibit yeast growth, instead, they supplied nutrients that may support proliferation of yeasts and other spoilage microorganisms, particularly upon silo opening and aerobic exposure.

The response observed in silages treated with *L. buchneri* and IgY at 350 and 700 g t⁻¹ is likely associated with increased acetic acid concentrations and reduced yeast activity, which together limit nutrient losses during aerobic exposure. Similar improvements in aerobic stability have recently been reported with novel biotechnological fermentation enhancers under tropical conditions, supporting the relevance of alternative biological strategies for silage preservation (Blacio et al., 2025).

Collectively, these results demonstrate the dual functionality of IgY antibodies in sugarcane silage, acting to suppress fermentative yeast metabolism while indirectly supporting favorable fermentation dynamics. To our knowledge, this study represents the first reported application of IgY antibodies in silage science. The complete suppression within the detection limits of the method of yeast populations observed at inclusion levels of 350–700 g t⁻¹ underscores the potent antifungal effect of IgY and highlights its potential as a novel biological strategy for enhancing silage preservation. This immunological approach reduced ethanol production, preserved water-soluble carbohydrates, and enhanced aerobic stability by preventing yeast growth for up to 72 h after silo opening. Inclusion level of 350 g t⁻¹ was particularly effective, positioning IgY as a biologically targeted and scalable strategy for improving the fermentative profile, nutritional quality, and post-ensiling stability of sugar-rich tropical silages.

Notably, at the highest inclusion level (700 g t⁻¹), a slight rebound in ethanol production accompanied by modest reductions in acetic and lactic acids was observed. This phenomenon may hypothetically reflect off-target or non-specific interactions of IgY antibodies with other microbial populations, including lactic acid bacteria, temporarily affecting their metabolic activity. Such non-specific binding events, while limited, can partially reduce acid accumulation and allow minor ethanol formation, highlighting a nuanced effect of high-dose IgY beyond yeast inhibition. These observations reinforce the importance of dose optimization to balance effective yeast suppression with minimal unintended impacts on beneficial microbial activity, although this hypothesis warrants further microbiological investigation.

Although a direct economic analysis was beyond the scope of the present study, silages with improved fermentation quality, higher organic acid retention, and reduced dry matter losses are expected to enhance nutrient availability and feed efficiency, with potential benefits for animal performance. Functionally, the ability of IgY to bind yeast surface antigens during the initial phase of ensiling suggests that antibody–antigen interactions interfere with microbial adhesion and establishment, a prerequisite for proliferation. Unlike conventional microbial inoculants, which rely on metabolic activity and competitive growth, IgY antibodies act through a non-metabolic mechanism, offering a strategic advantage for silage preservation by directly targeting spoilage organisms independently of environmental or microbial constraints.

### 4.1 Limitations and future perspectives

The present study provides evidence supporting the use of anti-yeast IgY antibodies as a novel biological additive for sugarcane silage, however, some limitations should be acknowledged.

First, the stability and functional activity of IgY antibodies throughout the entire 114-day ensiling period were not directly measured. Although fermentation outcomes clearly indicate effective yeast suppression, particularly at inclusion levels ≥ 350 g t⁻¹, the persistence of active antibody fractions during long-term storage remains to be elucidated. It is important to note that IgY activity is primarily required during the initial phase of fermentation, when pH remains relatively high and yeast populations are metabolically active. Because IgY activity is primarily required during the initial phase of fermentation, prolonged stability throughout the entire storage period is not essential for functional efficacy. Nevertheless, future studies should directly quantify antibody integrity and binding capacity during ensiling to further characterize its temporal dynamics.

Second, the experimental validation was conducted using a single forage species under controlled conditions. Sugarcane was intentionally selected because of its high concentration of water-soluble carbohydrates and its well-documented susceptibility to ethanol losses driven by fermentative yeasts. Compared with most temperate and tropical forages, sugarcane presents a particularly challenging fermentation environment, making it a robust model to test anti-yeast strategies. However, evaluation of IgY-based additives in other sugar-rich or high-moisture forages would broaden the understanding of its applicability across diverse production systems.

Third, the absence of in vivo animal performance validation represents an important next step in the translational development of this technology. While improvements in fermentation profile, dry matter recovery, and aerobic stability strongly suggest enhanced nutrient preservation, direct assessment of intake, digestibility, feed efficiency, and animal performance is required to establish cost–benefit relationships under practical conditions. Animal trials are therefore planned as a subsequent phase, which will require larger-scale production of the IgY additive and evaluation under commercial feeding scenarios.

Economic considerations are central to the feasibility of IgY-based silage additives. A detailed cost–benefit analysis was beyond the scope of the present study; however, several contextual factors support its potential viability. Regions with integrated sugarcane and poultry production systems offer logistical advantages for biological IgY production, including local availability of raw materials and established infrastructure for egg-derived products. In ruminant production systems, the use of whole egg-derived IgY preparations may reduce processing costs, as extensive purification steps are not required for rumen applications. Furthermore, alternative production strategies, such as recombinant expression systems or targeted peptide synthesis following antibody mapping, may further optimize scalability and cost efficiency in the future. Importantly, the economic return of this technology should ultimately be evaluated not solely based on additive cost, but on its capacity to reduce fermentative losses, preserve nutrients, and improve animal performance, thereby offsetting investment through improved feed efficiency.

Beyond silage preservation, the technological platform explored here opens broader perspectives for microbiota modulation in animal production. The same immunological approach could be adapted to target specific microbial groups in different contexts, including strategies aimed at improving fermentation efficiency, reducing undesirable microbial metabolites, or serving as a complementary alternative to antibiotic growth promoters. Additionally, IgY formulations may be developed in different physical forms, including liquid or paste preparations, potentially simplifying application and reducing processing costs depending on the production system.

Collectively, these aspects represent natural next steps in the development of IgY-based silage technology rather than fundamental constraints of the present findings. The results presented here establish a proof-of-concept framework demonstrating that targeted antibody strategies can effectively modulate fermentation dynamics in sugar-rich tropical silages, supporting further research toward practical implementation and broader applications in ruminant production systems.

## 5 Conclusion

The present study demonstrates that yeast-targeting IgY antibodies effectively improve fermentation quality and aerobic stability of sugarcane silage. Inclusion of IgY at 350 g t⁻¹ suppressed yeast growth, preserved fermentable carbohydrates, enhanced lactic and acetic acid concentrations, and reduced ethanol production, contributing to lower dry matter losses. These results indicate that IgY has potential as a targeted biological silage additive. Future research should explore in vivo animal feeding trials, evaluate IgY combinations with other additives, and assess large-scale production feasibility to optimize cost-effectiveness and practical application.

## Financial support statement

This work was supported by the São Paulo Research Foundation (FAPESP), Brazil [grant number 2017/01646-2].

## Ethical approval and yeast strain selection

All experimental procedures involving animals were approved by the Ethics Committee for Animal Use of the Animal Science Institute – IZ/APTA/SAA (Approval No. 262-18). Yeast strains were selected based on their dominance in sugarcane silage and their capacity to ferment sugars and organic acids. Strains included Candida glabrata, Pichia kudriavzevii, Pichia manshurica, Saccharomyces cerevisiae, Schizosaccharomyces pombe, Torulospora delbrueckii, and Debaryomyces etchellsii. All strains were registered in the Brazilian National System for Genetic Resource Management (SISGEN) prior to use.

## CRediT authorship contribution statement

**Balieiro G․N․:** Writing – review & editing, Writing – original draft, Validation, Supervision, Project administration, Methodology, Investigation, Funding acquisition, Formal analysis, Data curation, Conceptualization. **Rosa C․A․:** Investigation. **Engracia Filho J․R․:** Writing – original draft, Methodology, Investigation, Formal analysis, Conceptualization. **Budino F․E․L․:** Writing – original draft, Supervision, Investigation, Formal analysis. **Freitas A․W․P․:** Writing – original draft, Investigation. **Moraes J․E․:** Supervision, Project administration, Investigation, Data curation. **Pizzolante C․C․:** Supervision, Investigation.

## Declaration of competing interest

The authors declare that they have no known competing financial interests or personal relationships that could have appeared to influence the work reported in this paper.
